# Integration of P-CuO Thin Sputtered Layers onto Microsensor Platforms for Gas Sensing

**DOI:** 10.3390/s17061409

**Published:** 2017-06-16

**Authors:** Lionel Presmanes, Yohann Thimont, Imane el Younsi, Audrey Chapelle, Frédéric Blanc, Chabane Talhi, Corine Bonningue, Antoine Barnabé, Philippe Menini, Philippe Tailhades

**Affiliations:** 1 CIRIMAT, Université de Toulouse, CNRS, INPT, UPS, 118 Route de Narbonne, F-31062 Toulouse CEDEX 9, France; thimont@chimie.ups-tlse.fr (Y.T.); ielyounsi@gmail.com (I.e.Y.); bonning@chimie.ups-tlse.fr (C.B.); barnabe@chimie.ups-tlse.fr (A.B.); tailhades@chimie.ups-tlse.fr (P.T.); 2 LAAS-CNRS, Université de Toulouse, UPS, INSA, 7 avenue du colonel Roche, F-31031 Toulouse, France; chapelle@laas.fr (A.C.); frederic.blanc@laas.fr (F.B.); chabane.talhi@laas.fr (C.T.)

**Keywords:** gas sensor, RF sputtering, thin film, CuO, tenorite, photolithography, metal oxide microsensor, micro-hotplate, pulsed temperature

## Abstract

P-type semiconducting copper oxide (CuO) thin films deposited by radio-frequency (RF) sputtering were integrated onto microsensors using classical photolithography technologies. The integration of the 50-nm-thick layer could be successfully carried out using the lift-off process. The microsensors were tested with variable thermal sequences under carbon monoxide (CO), ammonia (NH_3_), acetaldehyde (C_2_H_4_O), and nitrogen dioxide (NO_2_) which are among the main pollutant gases measured by metal-oxide (MOS) gas sensors for air quality control systems in automotive cabins. Because the microheaters were designed on a membrane, it was then possible to generate very rapid temperature variations (from room temperature to 550 °C in only 50 ms) and a rapid temperature cycling mode could be applied. This measurement mode allowed a significant improvement of the sensor response under 2 and 5 ppm of acetaldehyde.

## 1. Introduction

Metal-oxide (MOS) gas sensors based on a micromachined silicon substrate [1] were a disruptive development which led to a mature and robust form of technology [2]. There are a few examples of devices on the market, which are notably based on SnO_2_ and WO_3_ metal oxides. To lower the resistivity of the sensitive film and improve the kinetics of the chemical reactions, commercial MOS gas sensors are operated in constant temperature mode (isothermal) knowing that the interactions between the sensitive material and the surrounding gases are temperature-dependent. Because the temperature dependence is not similar for all gases, the operation of a sensor at different temperatures can provide discrimination of several gases with one single sensor [3,4]. It has also been shown that with very short temperature pulses, transient sensor responses are strongly dependent on the ambient mixture of gases, which provides a good opportunity to enhance sensor selectivity [5,6,7,8]. These micro-hotplates can now be elaborated with different types of semiconducting sensitive layers, with the very interesting possibility of modulating the operational temperature and integrating the electronics with the sensor silicon chip. Current technologies allow temperature cycling up to several millions of cycles without failure. Despite their increased system-level complexity, microsensors have many advantages, such as, for example, high performance, small size, low cost, and low power consumption [9]. The latter requires on the order of a few or tens of mW for continuous operation, but sub-mW consumption can be reached by using a pulsed operating temperature [3]. Such microsensors are particularly suitable for air quality control systems in automotive cabins.

The literature therefore shows many examples of microsensors onto which sensitive layers have been deposited by using various methods, such as for example micropipetting [10,11,12,13], sputtering [14,15,16,17,18,19,20,21], precipitation–oxidation [22,23], stepwise-heating electrospinning [24], flame spray pyrolysis [25], spin coating [26], carbo-thermal route [27], evaporation [28], metal-assisted chemical etching [29], or organic binder printing [30]. Radio-frequency sputtering is a method compatible with the industrial fabrication of miniaturized sensors by microelectronics and MEMS (microelectromechanical systems) technologies. Radio-frequency (RF) sputtering has many other advantages, like the possibility of obtaining thin films with nanometric-scale grain sizes and very easy control of the inter-granular porosity by varying the deposition parameters [31,32]. Such films with controlled nanostructure are of great interest as sensitive layers [33,34,35] and can be integrated in gas sensing devices.

Cupric oxide (copper(II) oxide: CuO) is an intrinsically p-type semiconductor [36,37]. Among all other p-type semiconducting oxides it is the most studied for gas sensing applications [38] due to its low-cost, high stability and non-toxicity. Many researchers have focused on the development of novel CuO nanostructures for the detection of a large range of gases, such as for example organic gases [39], hydrogen sulfide (H_2_S) [40,41,42,43,44,45,46], CO [47,48,49,50], NO_2_ [50,51], ethanol gas [52,53,54], or NH_3_ [55].

In this work, we show the interest of using fully compatible micromachining technologies to elaborate microheaters and deposit CuO-sensitive layers to obtain sensors at the micronic scale. Elaboration of micro-hotplates, as well as photolithographic steps for layer integration, were carried out using the micromachining facilities of the Laboratory for Analysis and Architecture of Systems (LAAS-CNRS). The sensor is based on a p-type CuO resistive layer that was deposited by radio-frequency (RF) sputtering in the Interuniversity Center of Materials Research and Engineering (CIRIMAT). The microsensors were tested with variable thermal sequences with CO, NH_3_, C_2_H_4_O, and NO_2_, which are among the main pollutant gases found in automotive cabins. Many works have focused on the sensing properties of pure or doped CuO as the main sensitive material or as an additive for other semiconducting oxides. There are fewer articles related to the sensing properties of CuO layers integrated on microhotplates. For example, Walden et al. [56] tested inkjet-printed CuO layers for NH_3_ detection in rapid temperature cycled mode. Kneer et al. [57] used similar inkjet-printed CuO nanoparticles deposited on a microsensor and obtained good H_2_S selectivity in the NO_2_, NH_3_ and SO_2_ atmosphere but with a time pulsation of few minutes. However, there are no articles relating to acetaldehyde detection with a CuO-sensitive layer deposited on a microsensor and operated in pulsed temperature mode, although temperature cycling gives good results for acetaldehyde detection with other sensitive oxides [58].

## 2. Experimental

Thin sensitive films were deposited with an Alcatel SCM 400 apparatus using sintered ceramic targets of pure CuO with a relative density around 75% (10 cm in diameter). The RF power was lowered at 50 W to avoid target reduction [59] and the pressure inside the chamber was lower than 2 × 10^−5^ Pa before deposition. During the deposition of the films, the target-to-substrate distance was fixed at 7 cm (Table 1). The thicknesses of the deposited films were set to 50 nm on microsensors and 300 nm for the structural characterizations on fused silica substrates. In the case of copper oxides that can have multiple valences of copper, like in tenorite CuO (Cu^II^), paramélaconite Cu_4_O_3_ (mixed Cu^I^/Cu^II^) or cuprite Cu_2_O (Cu^I^), high deposition pressure could lead to a reduction [60] of the CuO target and the deposition of a phase with lower valences states. Moreover, as the layer had to be integrated by a wet process, a low deposition pressure of 0.5 Pa was preferred to obtain dense [61] oxide layer. These deposition conditions are then adequate to avoid the filling of the intergranular porosity of the sensitive layer with dye or any residue obtained during photolithographic process but should lead to layers with not optimized sensitivities. 

Thickness calibrations were performed with a DEKTAT 3030ST profilometer. The structure properties were determined by grazing incidence X-ray diffraction (GI-XRD) using a Bruker-AXS D8-Advance X-ray diffractometer equipped with a copper source (λCuK_α1_ = 1.5405 Å and λCuK_α2_ = 1.5445 Å) at 1° incidence, a Göbel mirror and Bruker LynxEye detector used in 0 D mode. The GI-XRD data were analyzed with the Bruker-EVA software and the JC-PDF database, and refined with the Rietveld method implemented in the FullProf-Suite program. Raman spectra were collected under ambient conditions using a LabRAM HR 800 Jobin Yvon spectrometer with a fiber coupled 532-nm laser. Spectra acquisition was carried out for 150 s using a ×100 objective lens and 600 gr/mm grating. During the measurement, the resulting laser power at the surface of the sample was adjusted to 1.7 mW. Examination of multiple spots showed that the samples were homogeneous. Microscopic studies were realized with a Veeco Dimension 3000 Atomic Force Microscope (AFM) in tapping mode equipped with a super sharp TESP-SS Nanoworld tip (nominal resonance frequency 320 KHz, nominal radius curvature 2 nm). The scanning rate was fixed at 1 Hz (1000 nm/s).

For sensing measurements, the sensors were placed into a chamber flown by different gases. The composition and humidity of the gas mixture were controlled by mass flow controllers (MFC). The heating and the sensing resistors of each sensor were connected to a source measurement unit (SMU). The whole test bench was automatically controllable thanks to a suitable interface and dedicated software. After a period of stabilization of 2 h under synthetic air, the target gases were introduced alternatively. The global flow (200 sscm) and the relative humidity (30%) remained constant during both air and target gas sequences. Response of gas sensor toward the four gases (CO, NH_3_, C_2_H_4_O and NO_2_) has been calculated according to the formula (1).

(1) $$S(\%)=\left(\frac{R_{gas}-R_{air}}{R_{air}}\right)\times 100$$

where, *R_air_* and *R_gas_*, are resistances in air and test gas, respectively.

## 3. Preparation of Microheaters

**Figure 1:**
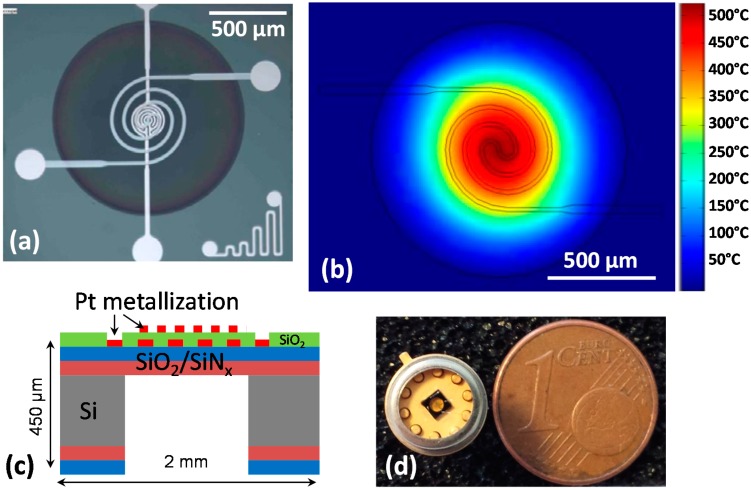
(**a**) Top view of the micro hotplate elaborated onto a membrane; (**b**) thermal simulation made with Comsol Multiphysics; (**c**) schematic view of a platform; (**d**) sensor packaged on TO5 housing.

The devices have been developed on an optimized microheater that can work at high temperature and low power consumption (500 °C and ~55 mW, respectively). In order to avoid edge effects, circular membrane geometry (Figure 1a) was chosen. Figure 1b shows the resulting thermal distribution simulated by Comsol Multiphysics software in such geometry. It can be observed that the temperature is homogeneous in the center of the heated area onto which the measurement electrodes are placed (Figure 1a).

**Figure 2:**
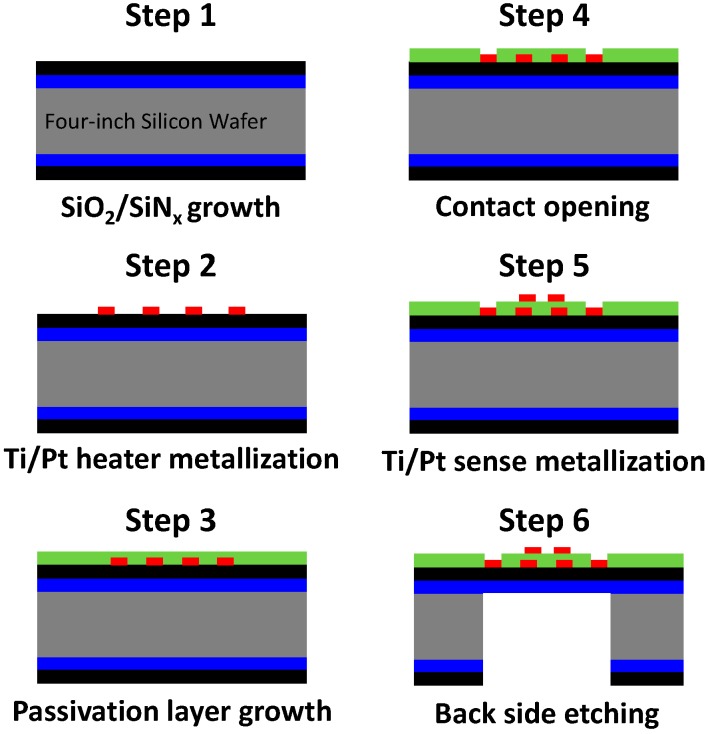
Main steps of platform elaboration process.

Micro-hotplates have been elaborated by photolithographic process. The detailed microfabrication steps are presented in Figure 2. The platform consists of a silicon bulk on which a thermally resistive bilayer SiO_2_/SiN_x_ membrane was grown. Afterwards, Pt metallization was carried out by lift-off process to obtain the heating resistor. A passivation layer was then deposited (a 0.7-µm thin PECVD SiO_2_ layer) and contacts were opened. Finally, a new lift-off step was used to elaborate the electrodes necessary for measuring the resistance of the sensing layer, and the rear side of the bulk was etched to release the membrane in order to increase the thermal resistance and then to limit thermal dissipation. Figure 1a shows the top view of the final membrane. Figure 1d shows a sensor mounted in its housing (TO5). 

Thermal measurement of the surface of the platform performed with a Jade MWIR infra-red camera (CEDIP) allowed the calibration between the power applied and the resulting heating temperature onto the membrane. The results given in the Table 2 show a good linear relation between the power applied and the temperature measured. The heating platform makes it possible to heat from room temperature to 550 °C in 50 ms and the cooling time is of the same order of magnitude [62]. This type of platform can thus generate very rapid temperature variations, which is suitable for operating the sensor in pulsed mode. At the end of the step 6 and before dicing the chips, it is possible to locally deposit a metal-oxide layer onto the electrodes to form the sensing thin film resistor. This will be described below in the Section 4. 

## 4. Integration of P-Type CuO Layer by Photolithography Process 

### 4.1. Structural Characterizations of CuO Layer

**Figure 3:**
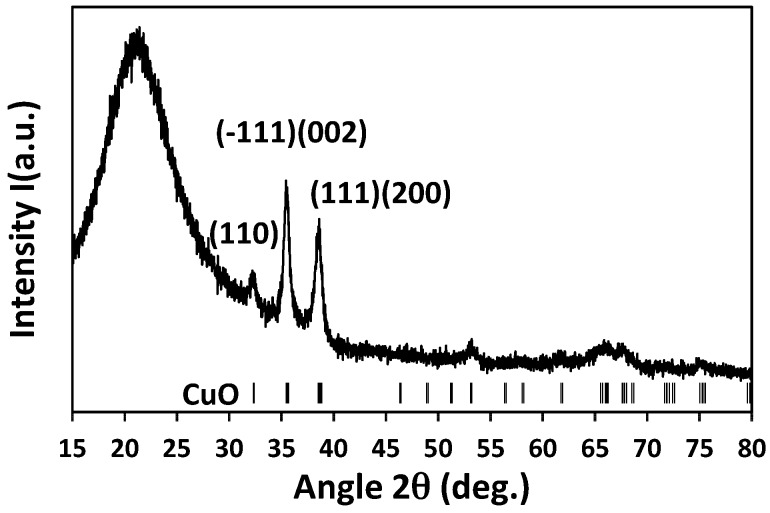
X-ray diffraction (XRD) pattern of CuO thin film annealed at 500 °C (thickness = 50 nm).

Figure 3 shows XRD pattern of copper oxide thin film deposited on a fused silica substrate and annealed at 500 °C. Measurement was carried out with a 50-nm-thick sample, similar to that deposited on the microsensor for gas sensing tests. The X-ray diffractogram clearly shows the presence of a pure tenorite phase (CuO: JCPDF 45-0937). The XRD patterns do not show any presence of extra phases with copper oxidation state lower than +II (like for example paramelaconite Cu_4_^II/I^O_3_, cuprite Cu_2_^I^O, or metallic copper Cu). 

**Figure 4:**
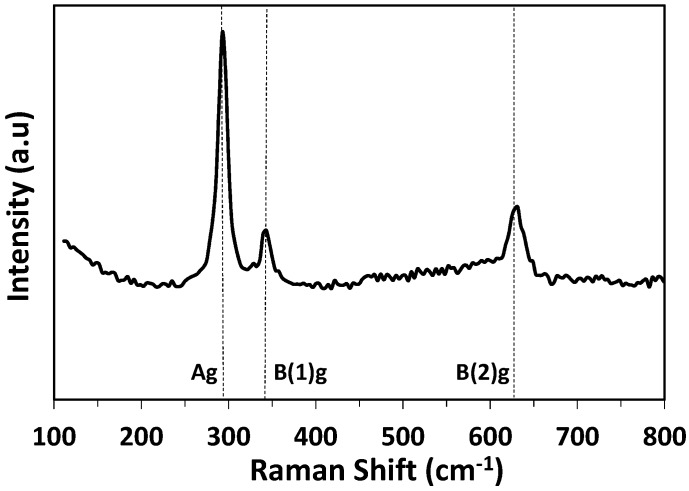
Raman spectra of CuO thin film annealed at 500 °C (thickness = 300 nm).

The Raman spectrum of a sample annealed at 500 °C is presented in Figure 4. The 50-nm-thick sample was too thin to be measured and a 300-nm-thick sample deposited with the same deposition conditions was characterized. Raman spectrum shows three vibration modes at 296, 346 and 636 cm^−1^, which are characteristic of the CuO phase [54,63,64] and can be attributed to Ag, B(1)g, and B(2)g modes, respectively. Raman spectra of the reduced phases containing Cu(I) such as paramelaconite Cu_4_O_3_ or cuprite Cu_2_O may be easily differentiated from those of tenorite phase CuO. In particular, paramelaconite provides a characteristic Raman peak at about 520–530 cm^−1^ and cuprite at 110 cm^−1^ and 220 cm^−1^. In the Raman spectra of as-deposited and annealed films, none of these signals are visible, confirming the absence of such phases.

In conclusion, the data obtained by XRD and Raman spectroscopy show a pure CuO phase in the deposited films.

**Figure 5:**
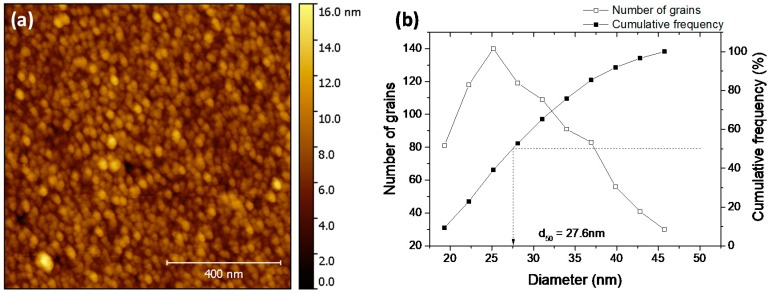
(**a**) Atomic force microscope (AFM) image of a 50-nm-thick CuO film annealed at 400 °C for 1 h under air atmosphere; (**b**) Grains size distribution deduced from the image analysis.

The image of the surface of CuO thin film, obtained by AFM, has been reported in Figure 5a. To ensure consistency with the layer used for sensing tests, a 50-nm thin film was observed. The surface consists of circular grains with surface domes (top of the grown column) which is a typical morphology in the case of the sputtered thin films. The distribution of the grains size shown in Figure 5b was estimated by an immersion threshold thanks to the Gwyddion software. The median grain size (d_50_) was found to be equal to 27.6 nm, which is close to the half thickness of the sample.

### 4.2. Description of the Integration Process

**Figure 6:**
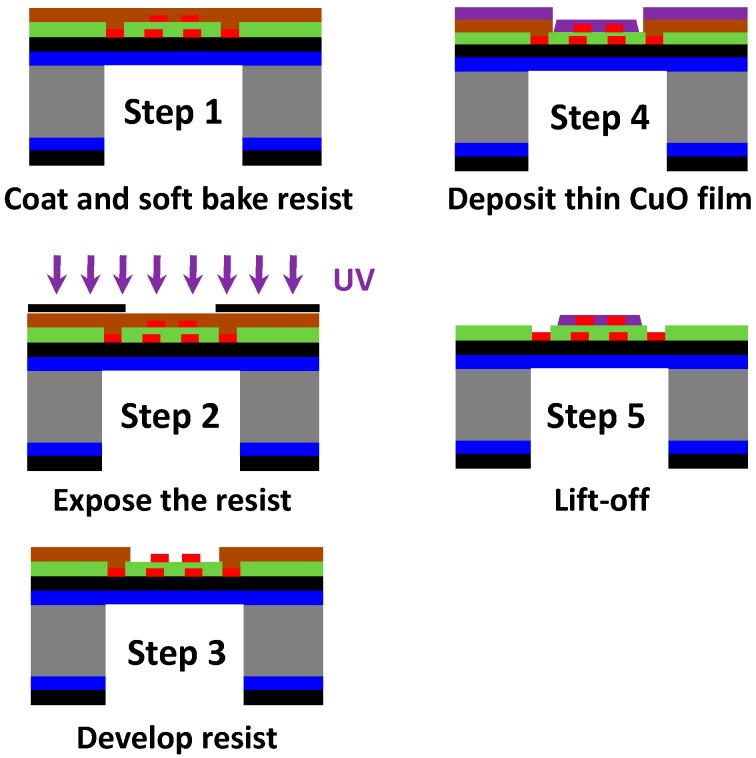
Main steps in the integration process of CuO-sensitive layers.

The integration of the copper oxide layer was performed using a classical photolithographic process (Figure 6). The lift-off resist was deposited in step 1. In the second and third steps the photoresist layer was exposed and then developed. The deposition of the 50-nm-thick CuO layer was undertaken using RF sputtering in step 4. Finally, all the unwanted parts were removed in step 6 by dissolution of the resist, thus leaving the sensitive layer in the desired areas.

**Figure 7:**
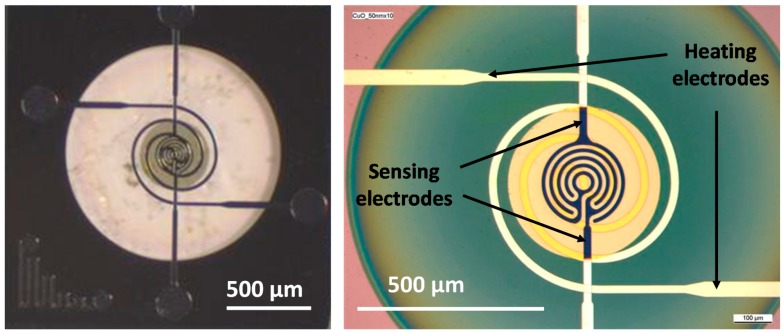
Images obtained by optical microscopy of a micro heater coated in the center with a p-type CuO semiconducting layer.

The deposition of the sensitive layer is a critical step, as the bombardment occurring during the sputtering process is able to damage the photoresist used to mask the part that does not have to be covered by the oxide layer. Figure 7 shows the successful integration of copper oxide onto a microheater. The diameter of the area covered by the sensitive CuO layer is around 400 µm. The diameter of the electrodes used for the electrical measurement was approximately 200 µm, which was then totally covered by the sensitive layer. 

## 5. Sensing Tests

**Figure 8:**
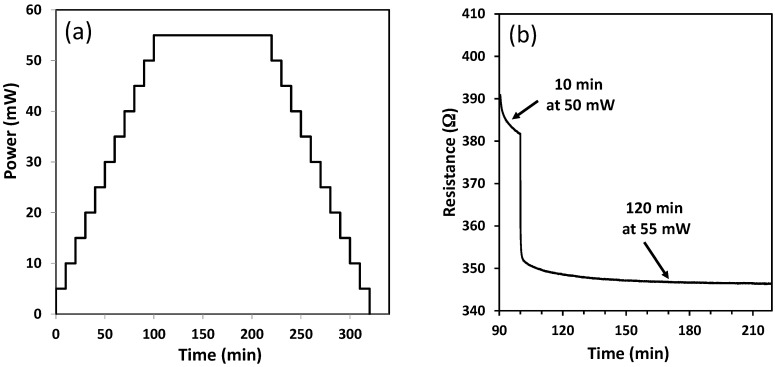
(**a**) Initialization program and (**b**) variation of the electrical resistance of the sensitive layer during the initialization (zoom in the heating power range of 50–55 mW, ~450–500 °C).

At first, the sensing device with integrated CuO layer (as-deposited) was annealed from 0 mW (room temperature) to 55 mW (~500 °C) in step at 5 mW/10 min and kept for 120 min at 55 mW to stabilize both the sensing layer and the microheater (Figure 8). XRD showed (Figure 3) that the CuO phase is stable up to 500 °C, and then only microstructural reorganization and a possible formation of slight over-stoichiometry in copper oxide (CuO_1+δ_) are expected. 

Figure 8b shows the evolution of the layer resistance during initialization in the 50–55 mW heating power range. A decrease of the resistance was observed during annealing with 50 and 55 mW heating power. After 120 min at 55 mW the resistance of the sensitive layer was stabilized.

The microsensor based on the CuO semiconducting layer (thickness 50 nm) was tested under carbon monoxide CO, ammonia NH_3_, acetaldehyde C_2_H_4_O, and nitrogen dioxide NO_2_ according to the gas concentrations shown in Table 3. Each gas concentration was chosen close to the threshold concentration given by the various national (ANSES, French Agency for Food, Environmental and Occupational Health and Safety) [65] and international (WHO, World Health Organization) [66] health-based guidelines and guidance values for short time exposure in the case of indoor polluting gases. This is the reason why the concentration ranges are different for the four target gases.

**Figure 9:**
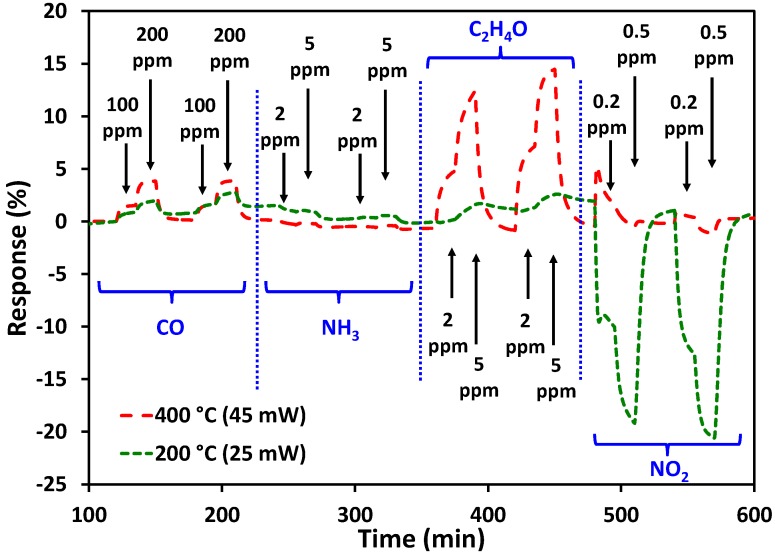
Response of the CuO-sensitive layer at 400 °C and 200 °C with constant temperature mode.

In a first step, a “classical” constant temperature profile has been used. In this case the temperature is maintained at a constant temperature, while the gas composition and its concentration are alternated. The Figure 9 shows the response obtained at 400 °C (45-mW heating power) and at 200 °C (25-mW heating power) with the gases and the concentrations presented in the Table 3. Before starting the gas alternation, the resistance was stabilized under air for 2 h. For each gas, two concentrations were used for 15 min, and this sequence was repeated twice. Before changing the composition of the gas, the sensor was returned to air for 30 min. 

The CuO layer showed decreases in resistance upon exposure to oxidizing gas (NO_2_) and increases in resistance upon exposure to reducing gases (C_2_H_4_O, NH_3_, and CO). This is consistent with the p-type semiconducting behavior of copper oxide. The results show that the response of the sensor is very low for carbon monoxide and almost zero for ammonia. On another hand, significant response values were obtained for acetaldehyde at 400 °C and for nitrogen dioxide at 200 °C. It can be noted in contrast that the response of the CuO layer under acetaldehyde at 200 °C and under nitrogen dioxide at 400 °C is roughly equal to zero. 

These results are in accordance with the bibliography which shows that the sensitivity of CuO toward C_2_H_4_O and NO_2_ in constant temperature mode is dependent on the temperature measurement. For NO_2_ gas sensing, various studies [50,57,67] have shown that higher sensitivity is obtained at low or moderate temperature (150–250 °C). For acetaldehyde it is more difficult to refer to sensing studies as there is no article related to the detection of this gas by CuO. However, Cordi et al. [68] have carried out temperature-programmed oxidation (TPO) measurements on CuO for catalytic applications, and they showed that C_2_H_4_O is oxidized at higher temperatures (340 °C). By alternating high and low measurement temperature, the discrimination of C_2_H_4_O and NO_2_ can then be improved. Even if the response values remain quite low, it should be borne in mind that the gas concentrations used for the test are also very low.

**Figure 10:**
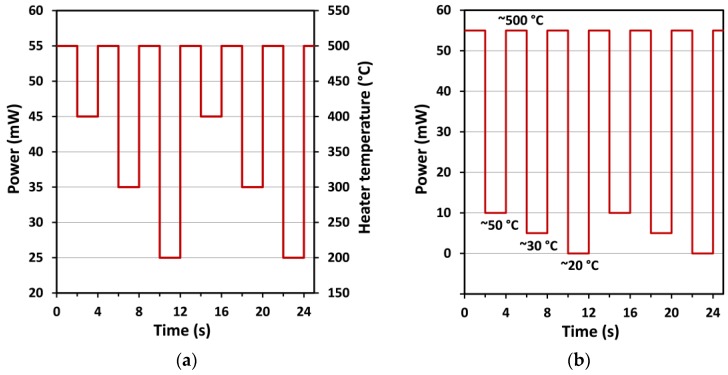
Power profiles in (**a**) high temperature range and (**b**) low temperature range.

In a second step, a dynamic test profile was carried out with the same gas and concentrations. Many works have already shown the interest of operating the sensor with temperature cycling by using different profile shape and plateau duration in order to rapidly change its sensitivity and then the selectivity after suitable data treatment. Among the thermal cycle approaches, one consists of making the temperature vary with stair shape from ambient to high temperature or vice-versa. Another consists of using short heating or cooling pulses from a reference temperature which can be set at ambient, high or intermediate temperature [69]. The pattern we chose in this study is the latter, with two-second steps at each target temperature and a baseline fixed at 500 °C. The measurement pattern had already been optimized in the past and the temperature profile that allowed a good reproducibility, the fastest stabilization, and the best discrimination was selected [70]. The short plateau duration is well-adapted to only observe transient phenomena, while the high baseline temperature allows regular cleaning of the surface of the sensitive layer to obtain good reversibility and reproducibility. Moreover, this profile is easy to implement in embedded electronics. The temperature profiles are presented in the Figure 10a,b: each step lasts 2 s; the temperature baseline is the highest operating temperature (500 °C), while the other steps are at lower temperatures (400, 300, and 200 °C for high temperature measurements and 50, 30, and 20 °C for low-temperature measurements), and a complete cycle lasts 12 s. This profile is repeated throughout the test under various gaseous atmospheres.

**Figure 11:**
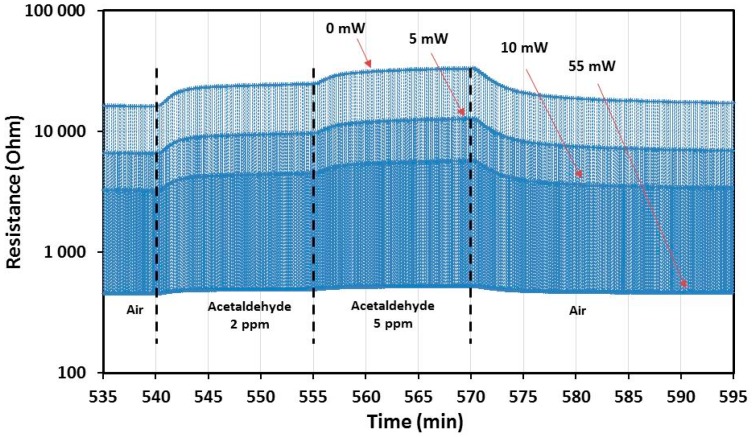
CuO sensor operated with dynamic temperature cycling mode (temperature cycle of the Figure 10b has been used). Resistance measurement under 30% RH for 1 h with a first injection of 2 ppm of acetaldehyde (15 min), then a second injection of 5 ppm (15 min). The resistance values corresponding to the four temperatures (or heating power) are multiplexed due to two-second step temperature cycling.

The Figure 11 shows an example of resistance measurement under pulsed temperature mode over one hour with a relative humidity (RH) of 30%. The sensor has been set successively under four different “ambiances”: (1) synthetic air; (2) 2 ppm of acetaldehyde; (3) 5 ppm of acetaldehyde; and finally (4) synthetic air. During these “sequences”, the sensor is periodically powered (on the heater) with the four different two-second steps (0, 5, 10 and 55 mW) as seen before in Figure 10b, considering that 55 mW is the reference or the base-line. In this view the resistance values at all temperature steps are multiplexed, and it is hardly possible to guess all the step transitions and the behavior of the resistance at each power step. For this reason, it is necessary to extract or separate results from the different power-steps in order to calculate their associated normalized response in the same way as it was done for the static operating mode. 

**Figure 12:**
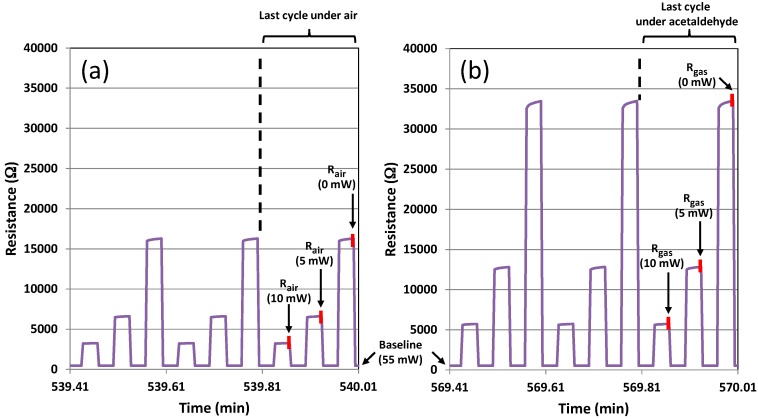
Detailed view of resistance variation during (**a**) the last three cycles under air and (**b**) the last three cycles under 5 ppm of acetaldehyde. The sensor was operated with dynamic temperature cycling mode (the sequence presented in the Figure 10b has been applied), under 30% relative humidity, and a 55 mW baseline. *R_air_*: resistance in air; *R_gas_*: resistance in test gas.

The detailed views of the resistance variation under the same pulsed temperature cycles are shown in Figure 12a for the last three cycles just before the injection of acetaldehyde and in Figure 12b for the last three cycles at the end of the gas sequence under 5 ppm of acetaldehyde. The small red lines correspond to the penultimate point of each step in the last cycle before a gas transition. For the calculation of the response ∆R/R given in the Formula (1), the reference values (*R_air_*) have been taken in the last cycle under air for each heating power, while the *R_gas_* values have been taken in the last cycle under target gas (acetaldehyde in the example of the Figure 12). 

**Figure 13:**
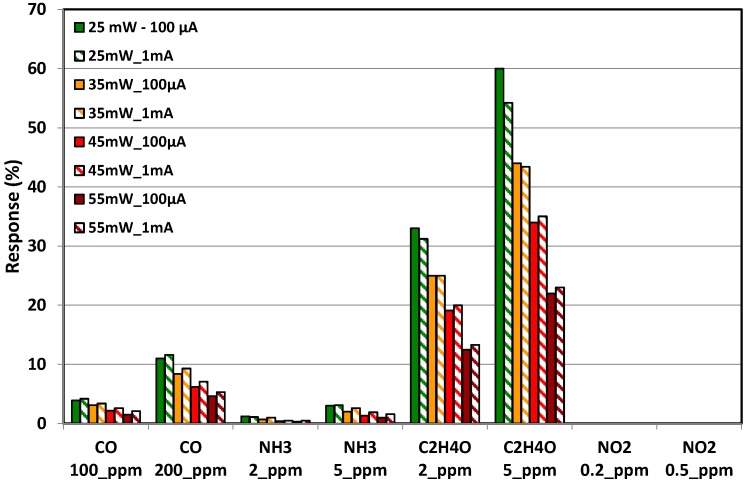
Comparison of the response obtained under dynamic tests at high temperature range (200–500 °C) by using two different bias currents (100 µA and 1 mA).

Pulsed temperature cycling mode has been carried out under the same gases and concentrations which were used in static mode previously (Table 3), during 15 min for each target gas and with 30% RH. The response has been calculated according to the procedure explained just before. The sequence from the Figure 10a was used first. Every 12 s (i.e., one complete cycle) the power was switched between 100 μA and 1 mA to study, in addition to the gas pulse, the influence of the current applied during the electrical resistance measurement. Because all this information was multiplexed in the same experimental file, the normalized responses for each gas, concentration and current were extracted and presented as a synthetic result in the form of bar-graph in the Figure 13. It can be seen that the values of the bias current have almost no influence on the response. On the contrary, the measurement temperature has a strong effect and it can be seen that the best results have been obtained for the lower power step (25 mW~200 °C). Even if the response under CO and NH_3_ has been slightly increased in dynamic mode, the maximal change in resistance remains less than about 10% for 200 ppm of CO and 5% for 5 ppm of NH_3_. The strongest improvement has been obtained for the measurements under C_2_H_4_O, which show an improvement of the response that has been multiplied by 4 in comparison with the tests carried out with constant temperature profiles. Moreover, the trend is reversed between the two measurement modes. In constant temperature mode the response decreases when the measurement temperature (i.e., the heating power) is lowered, whereas the response increases strongly in dynamic mode. 

The use of a pulsed-temperature operating mode promotes the transient chemical reactions. The difference observed between constant and modulated temperature mode can be due to the fact that the adsorption/desorption and reaction phenomena occur in out of equilibrium conditions in this last mode [71]. The effective adsorption, which is the result of the competition between OH^−^, O_2_^−^, O^2−^, O^−^ and the target gas, is thus modified when the sensitive layer is brought to high temperature and then cooled very rapidly [72]. Another consequence of this rapid thermal cycling mode is the total disappearance of the resistance variation under NO_2_, which is not observed even at low heating power. It has already been shown in the literature that NO_2_ has a complex interaction with oxide surface [73] which can lead in some cases to a transition from reducing to oxidizing behavior with the operating temperature [74]. The lack of response of NO_2_ on the surface of CuO in quick pulsed temperature mode can be due to kinetic reactions longer than pulse duration and/or opposite reactions that counteract each other in the high and low temperature alternate steps.

**Figure 14:**
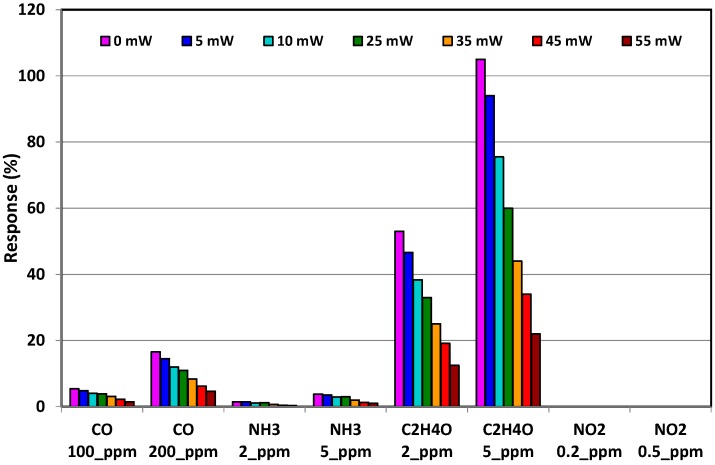
Comparison of the response obtained under dynamic tests in the full temperature range (20–500 °C). Bias current has been fixed at 100 µA.

To further explore the effect of the decrease in the measurement temperature, three additional powers (10, 5 and 0 mW) have been used according to the cycle shown in Figure 10b. The comparison of all measurements carried out between 20 °C (0 mW) and 500 °C (55 mW) with 100-µA bias current are shown in the Figure 14. The results show that the increase of the response observed when the measuring heating power was decreased from 55 mW to 25 mW continues to occur when it is lowered down to 0 mW. Finally, the response under C_2_H_4_O could be multiplied by 7 in comparison with constant temperature mode when a thermal cycling mode was applied. This last measurement also confirms that cycled mode allows total selectivity with respect to NO_2_, which is not detected whatever the temperature applied. 

## 6. Conclusions

Micro-hotplates were first prepared by using silicon microtechnologies. P-type semiconducting CuO layers deposited by RF sputtering were integrated onto these microsensors by using classical photolithography technologies that were used for the preparation of micro-hotplates. Even if this route has the disadvantage of exposing the sensitive layer to chemical products which are able to attack it (acidic or basic solutions), the integration of the copper oxide layer could be successfully carried out. Because the microheater was designed on a membrane, it was then possible to generate very rapid temperature variations and a rapid temperature cycled mode could be applied. This measurement mode showed a strong improvement, by of a factor of 7, in the sensor response under 2 and 5 ppm of acetaldehyde and by a factor 2 in the case of carbon monoxide. 

## Figures and Tables

**Table 1:** Deposition parameters of thin sensitive films.

Target material	CuO
Magnetron	Yes
Substrates	Fused silica and micro-hotplate
Power	50 W
Argon pressure	0.5 Pa
Target to substrate distance	7 cm
Deposition rate	6.1 nm/min

**Table 2:** Temperature reached in the center of the center of the microheater vs applied heating power.

Power (mW)	Temperature (°C)
55	500.7
45	402.7
35	304.8
30	255.8
25	206.9

**Table 3:** Gases and concentrations used during the sensing tests with the CuO-sensitive layer.

Gas	Concentration (ppm)	
CO	100	200
NH_3_	2	5
C_2_H_4_O	2	5
NO_2_	0.2	0.5
